# Potential for false positive HIV test results with the serial rapid HIV testing algorithm

**DOI:** 10.1186/1756-0500-5-154

**Published:** 2012-03-19

**Authors:** Steven Baveewo, Moses R Kamya, Harriet Mayanja-Kizza, Robin Fatch, David R Bangsberg, Thomas Coates, Judith A Hahn, Rhoda K Wanyenze

**Affiliations:** 1 Department of Medicine, Makerere University School of Medicine, Kampala, Uganda; 2 Department of Disease Control and Environmental Health, Makerere University School of Public Health, Kampala, Uganda; 3 Department of Medicine San Francisco General Hospital, University of California San Francisco, San Francisco, California, USA; 4 Massachusetts General Hospital Center for Global Health and Harvard Medical School, Boston, Massachusetts, USA; 5 Division of Infectious Diseases, David Geffen School of Medicine, University of California Los Angeles, Los Angeles, California, USA

**Keywords:** False positive, HIV testing algorithm, Rapid diagnostic tests, Qualitative PCR testing

## Abstract

**Background:**

Rapid HIV tests provide same-day results and are widely used in HIV testing programs in areas with limited personnel and laboratory infrastructure. The Uganda Ministry of Health currently recommends the serial rapid testing algorithm with Determine, STAT-PAK, and Uni-Gold for diagnosis of HIV infection. Using this algorithm, individuals who test positive on Determine, negative to STAT-PAK and positive to Uni-Gold are reported as HIV positive. We conducted further testing on this subgroup of samples using qualitative DNA PCR to assess the potential for false positive tests in this situation.

**Results:**

Of the 3388 individuals who were tested, 984 were HIV positive on two consecutive tests, and 29 were considered positive by a tiebreaker (positive on Determine, negative on STAT-PAK, and positive on Uni-Gold). However, when the 29 samples were further tested using qualitative DNA PCR, 14 (48.2%) were HIV negative.

**Conclusion:**

Although this study was not primarily designed to assess the validity of rapid HIV tests and thus only a subset of the samples were retested, the findings show a potential for false positive HIV results in the subset of individuals who test positive when a tiebreaker test is used in serial testing. These findings highlight a need for confirmatory testing for this category of individuals.

## Background

Rapid HIV tests provide quick results [1], usually in less than 30 minutes, can be performed by any adequately trained health worker, do not require sophisticated equipment and can be stored at room temperature (20^°^C-30^°^C); [2]. Yet, their diagnostic accuracy has been found to be comparable to that of Enzyme Linked Immunosorbent Assay (ELISA) [1,3]. These advantages make the rapid HIV tests suitable for use in geographically remote areas with little or no laboratory infrastructure and in small hospitals that perform few HIV tests [4]. Determine and Uni-Gold have a sensitivity of 100% [5,6] and STAT-PAK is 99.7% [7] sensitive. The specificities for Determine, Uni-Gold and STAT-PAK are 99.87% [5], 99.7% [6] and 99.9% [7] respectively. Despite the high sensitivity and specificity, rapid diagnostic tests have a rare potential for false negative and false positive test results [8].

The World Health Organization HIV retesting guidelines [9] highlight the criteria for retesting persons with indeterminate and negative HIV test results in order to reduce the likelihood of false negative HIV results in early HIV infection [9]. However, these guidelines do not address the potential for false positive HIV results. This paper sought to identify the potential for false positive results in the serial HIV testing algorithm of Determine-STAT-PAK-Uni-Gold, specifically in the subset of individuals who tested positive on Determine, negative on STAT-PAK, and positive on Uni-Gold, by conducting further testing using Deoxyribonucleic acid Polymerase chain reaction Qualitative (DNA PCR QL) testing.

## Methods

### Study procedures

The study was conducted as part of the HIV VCT and Linkage to Care study in Mulago hospital [10]; a research collaboration between Makerere University, University of California Los Angeles (UCLA) and University of California San Francisco (UCSF).

Adult patients (≥ 18 years) who had never tested for HIV, or tested negative at least 1 year prior to recruitment and willing to receive a HIV test were eligible [11]. Ethical approval was obtained from the collaborating institutions' Ethical Review boards as well as Uganda National Council of Science and Technology and each participant provided verbal and written informed consent prior to enrolment into the study.

### Laboratory testing

**Figure 1:**
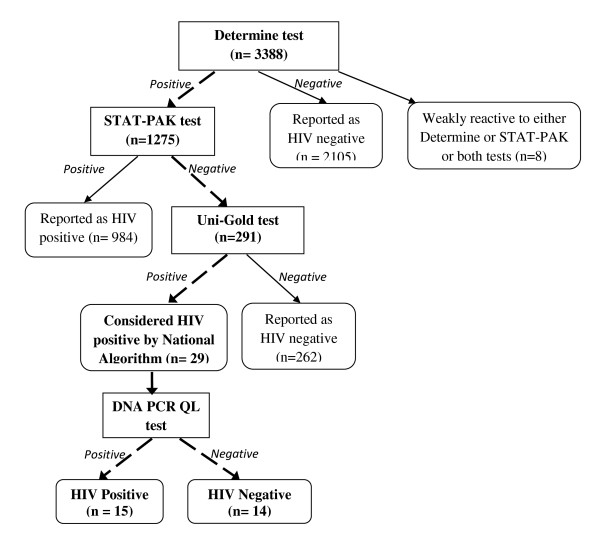
**Flow diagram of HIV testing algorithm**.

HIV counseling and testing was offered after enrollment and baseline interview. Following pre-test counseling, the counselor contacted the laboratory technician to draw blood for HIV testing [11]. HIV testing was done using the serial testing algorithm using the Determine (Abbott Laboratories, Abbott Park, IL), STAT-PAK (Chembio Diagnostics), and Uni-Gold™ Recombigen^® ^(Figure 1). Subjects that tested HIV negative on Determine were reported as negative. Subjects that tested positive on Determine, negative on STAT-PAK and negative on Uni-Gold were also reported as negative. According to the national algorithm, samples that tested positive on Determine, negative on STAT-PAK and then positive on a Uni-Gold (tie-breaker test) would be reported as positive and not retested. However, we conducted further testing of the latter samples using Deoxyribonucleic acid Polymerase chain reaction Qualitative (DNA PCR QL) tests (Ampiclor HIV-1 DNA PCR Test version 1.5) [12]. The 29 samples that tested positive with the tie breaker were not retested with rapid tests before the DNA PCR QL testing.

### Statistical analysis

Data captured in a Microsoft Excel spreadsheet was exported to SAS program for statistical analysis.

## Results

Overall, 3,388 individuals were tested for HIV between May 2008 and June 2011 and 1004 (29.6%) tested HIV positive (Figure 1). Twenty-four percent of men and 34% of women were HIV positive. Two thirds (62.1%, n = 2105) of individuals tested negative on Determine and were reported as negative. Of the 1275 that tested positive on Determine, 984 also tested positive on the STAT-PAK and were reported as positive, while 291 were negative on STAT-PAK and were retested with Uni-Gold. Of the 291 that were retested with Uni-Gold, 262 tested negative and were reported as negative; and 29 tested positive. The 29 samples were further tested using DNA PCR QL, and 14 (48.2%) tested HIV negative. DNA PCR has a very high specificity of 99.5%, [95% CI 99.1-99.9], and is thus an ideal choice for identifying false positive results [13,14]. Eight participants who were weakly reactive to the Determine and/or STAT-PAK test were also further tested using DNA PCR QL, but were not included among the false positive results in this study since they would have been retested with ELISA, according to the current national algorithm [15].

## Discussion

These findings show potential for false positive HIV test results in the subset of individuals who tested positive on Determine, negative on STAT-PAK and positive on Uni-Gold™. DNA PCR QL test may result in occasional false positives but not false negatives [8], which implies that the HIV negative DNA PCR QL results in this study are likely to be accurate. Misdiagnosis of HIV can lead to psychological difficulties and psychiatric morbidity, has public health and epidemiological implications and can lead to medico-legal conflict, and should thus be avoided to the extent possible [16].

This study is limited in that it was not primarily designed to evaluate the validity of rapid HIV test results. Since only individuals with positive tie-breaker tests were further tested using DNA PCR QL, we cannot estimate the overall false positive rate. However, these findings highlight an important subgroup of individuals that require confirmatory testing with DNA PCR or other highly specific test. There is need for further studies to examine the occurrence of false positive HIV test results and mechanisms for minimizing this challenge.

Conclusion Individuals who test positive on Determine, negative on STAT-PAK and positive on Uni-Gold should be considered inconclusive and be further tested using DNA PCR QL or other validated tests to minimize the occurrence of false positive HIV test results.

## Competing interests

The authors declare that they have no competing interests.

## Authors' contributions

Conceived, designed and performed the experiments; SB, MRK, HMK, RF, DBR, TC, HJA, RWK. Analyzed the data: SB, RF, JAH. Contributed reagents/materials/analysis tools: SB, MRK, HMK, RF, DBR, TC, HJA, RWK. Wrote the paper, proof read and approved the final manuscript: SB, MRK, HMK, RF, DBR, TC, JAH and RWK.
